# Graves on the Relapse Periods of Ague, and the Indications of Treatment

**Published:** 1846-07

**Authors:** 


*Graves on the Relapse Periods of Ague, and the Indications of Treatment.*—Having noticed with much anxiety and accuracy the course of a quartan ague for twenty-seven months, I constructed a table for the purpose of obtaining a connected view of the number and dates of the fits. This table had been made for some time before I discovered that it contained *data*, which authorize us in concluding that the law regulating the periodicity of agues applies not only to the succession of paroxysms, but is extended to the free intervals between them—in other words that the same law of periodicity which governs the disease, while it occasions fits, continues likewise to preside over its latent movements during the interval when no fit occurs, and thus the true periodic rate is carried on, though as in a clock from which the striking weight has been removed, the usual signal does not mark the termination of each certain definite portion of time.

This law, now for the first time brought to light, exhibits a new example of the tenacity with which periodicity clings to a disease, when once firmly impressed on it, and recalls a very similar phenomena observed with respect to the catamenia, which, having been suppressed for many months, not unfrequently reappears on the very day on which the monthly period should have occurred, had no such suppression taken place.

A knowledge of this law will, therefore, prove of the greatest importance, in enabling us to guard against the return of the disease; for, for several weeks after the series of fits has ceased, we can point out to the patient on what days they are liable to reappear; and, consequently, he can upon those days more effectually guard against the occasionally exciting causes of the disease, such as cold, fatigue, &c.

In reference to the treatment by quinine, the writer believes the practice generally followed to be wrong. He objects to the continued use of the remedy, as thus the constitution becomes accustomed to its influence when the ague-fit is absent, and then that influence is weakened.

The mode of treatment which I adopted was calculated to avoid this inconvenience. It consisted of giving the quinine for four successive days, and intermitting it for the six following days, thus embracing the interval comprehended in three fits. By these means it was hoped to keep the system sufficiently under the curative influence of the quinine while we avoided rendering the constitution too familiar with the medicine, the six-day interval preventing it from becoming saturated by the quinine.—*London Lancet.*

